# Integrated microbial and endocrine signatures of obesity: *Enterobacteriaceae* alterations, hormonal profiles, and dietary correlates in adult males

**DOI:** 10.3389/frmbi.2026.1867961

**Published:** 2026-09-09

**Authors:** Ranjeet Kumar Vishwakarma, Bhupendra Singh Yadav, Priyanka Gautam, Minakshi Sahu, Gopal Nath

**Affiliations:** 1 Department of Physiology, Faculty of Modern Medicine, Institute of Medical Sciences, Banaras Hindu University, Varanasi, Uttar Pradesh, India; 2 Department of Neurology, Faculty of Modern Medicine, Institute of Medical Sciences, Banaras Hindu University, Varanasi, Uttar Pradesh, India; 3 Department of Microbiology, Faculty of Modern Medicine, Institute of Medical Sciences, Banaras Hindu University, Varanasi, Uttar Pradesh, India

**Keywords:** body mass index, *Enterobacteriaceae*, ghrelin, insulin, leptin, obesity

## Abstract

**Introduction:**

Obesity is a multifactorial metabolic disorder increasingly associated with alterations in gut microbial composition and endocrine imbalance. In the present study, microbiological analyses were limited to culture-based characterization of facultative anaerobic *Enterobacteriaceae*. Members of the Enterobacteriaceae family, particularly *Klebsiella pneumoniae (Kpn)*, have been implicated in metabolic endotoxemia and inflammation; however, integrated data combining microbial profiling, metabolic hormones, and dietary patterns in Indian populations remain limited.

**Methods:**

This case-control observational study involved 49 males (27 obese, 22 normal-weight controls). Anthropometric data were collected, and fasting serum leptin, ghrelin, and insulin were measured via ELISA. Stool samples were cultured for *Enterobacteriaceae*, identified biochemically, and genotyped using ERIC-PCR. Dietary patterns were evaluated with questionnaires.

**Results:**

Obese participants showed significantly higher BMI, leptin (5.43±0.67 ng/mL), and insulin levels (3.21±0.27 mIU/L) than controls (p<0.05), while ghrelin levels did not differ significantly. Leptin positively correlated with BMI in obese individuals (r=0.423, p=0.028). Microbiological analysis yielded 82 isolates, with Kpn more prevalent among obese (92.86%) than controls (7.14%), whereas *Escherichia coli* predominated among controls (51.22%). ERIC-PCR demonstrated distinct genetic clusters among isolates. Obese participants also reported higher intake of fast food, refined carbohydrates, and soft drinks, correlating with elevated insulin levels.

**Conclusion:**

These findings suggest possible associations between Enterobacteriaceae prevalence, metabolic hormones, and dietary patterns in obesity and support microbiota-targeted and dietary interventions.

## Graphical Abstract

**Figure:**
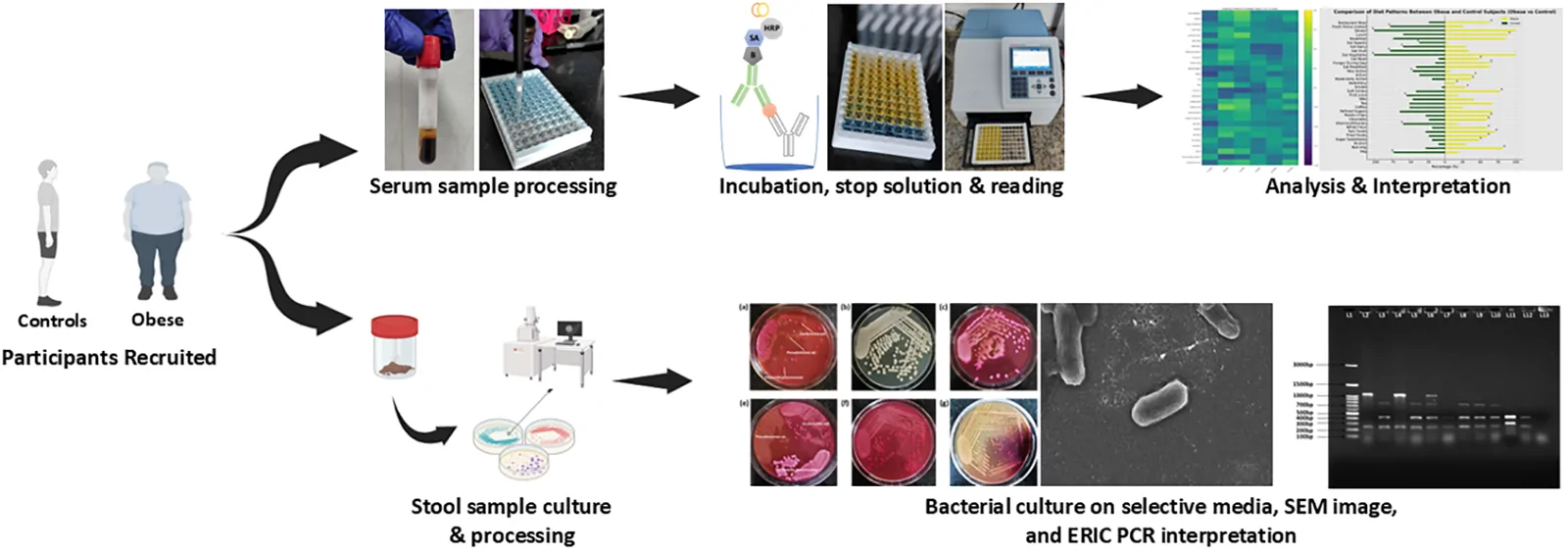

## 1 Introduction

Obesity has emerged as one of the most significant public health challenges of the 21st century, driven by rapid urbanization, sedentary lifestyles, and increased consumption of energy-dense, high-processed foods (Afshin et al., 2017). According to recent global estimates from the World Health Organization and the NCD Risk Factor Collaboration, more than one billion people worldwide are currently living with obesity, with prevalence rates nearly tripling in women and quadrupling in men since 1975 (Phelps et al., 2024). Projections suggest that by 2030, over 1.12 billion adults may be classified as obese, underscoring the urgency of understanding the complex biological mechanisms underlying this condition (Kelly et al., 2008; Vishwakarma et al., 2025a). Obesity is not merely an excess accumulation of adipose tissue; it is a chronic, relapsing metabolic disorder strongly associated with type 2 diabetes mellitus, cardiovascular diseases, non-alcoholic fatty liver disease, certain cancers, and increased susceptibility to infections (Vishwakarma et al., 2025a).

Traditionally, obesity has been explained by an imbalance between caloric intake and energy expenditure (Adamska-Patruno et al., 2018). However, emerging evidence indicates that this simplistic view does not fully capture the intricate network of hormonal, microbial, immunological, and metabolic factors involved. Among these, endocrine regulators such as leptin, ghrelin, and insulin play central roles in maintaining energy homeostasis (Klok et al., 2007). Leptin, a 16 kDa adipokine secreted primarily by adipocytes, acts on the hypothalamus to suppress appetite and increase energy expenditure. In individuals with obesity, circulating leptin levels are typically elevated, yet appetite suppression fails due to a phenomenon known as leptin resistance. This impaired signaling contributes to persistent hyperphagia, reduced energy expenditure, and progressive weight gain (Adamska-Patruno et al., 2018; Diwan et al., 2018).

Ghrelin, predominantly secreted by the stomach, functions as an orexigenic hormone that stimulates appetite and promotes food intake, with levels rising before meals and declining afterward (Adamska-Patruno et al., 2018; Korek et al., 2013; Rodríguez, 2014). Insulin, secreted by pancreatic β-cells, regulates glucose uptake and metabolism and exerts inhibitory effects on ghrelin secretion. In obesity, chronic low-grade inflammation and excess adiposity contribute to insulin resistance and compensatory hyperinsulinemia (Hardy et al., 2012). Importantly, leptin and insulin signaling pathways interact closely within the hypothalamus, and their dysregulation creates a vicious cycle of metabolic imbalance (Klok et al., 2007). Despite extensive research on hyperleptinemia and hyperinsulinemia in obesity, the upstream modulators of leptin resistance remain incompletely understood (Korek et al., 2013).

In recent years, the gut microbiota has emerged as a critical determinant of host metabolism and endocrine regulation (Vishwakarma et al., 2025a). The human gastrointestinal tract harbors approximately 10¹^4^ microorganisms, representing more than 1,000 bacterial species. The dominant phyla Firmicutes, Bacteroidetes, Actinobacteria, and Proteobacteria collectively influence nutrient absorption, short-chain fatty acid production, immune maturation, and energy harvesting (Aoun et al., 2020). Dysbiosis, characterized by altered microbial composition and reduced diversity, has been consistently associated with obesity and metabolic syndrome. Microbial metabolites can modulate inflammatory pathways, intestinal permeability, and adipokine secretion, thereby influencing systemic metabolic homeostasis (Vishwakarma et al., 2025a).

Within the Proteobacteria phylum, the family *Enterobacteriaceae* occupies a unique and clinically significant position. This family includes several Gram-negative, facultatively anaerobic enteric bacteria such as *Escherichia coli (E. coli), Klebsiella pneumoniae (Kpn), Enterobacter* spp., and *Citrobacter* spp (Gautam et al., 2026). While many members of the human gut microbiota are commensals, certain strains possess pathogenic potential and can disrupt intestinal homeostasis (Kumar et al., 2022). A defining feature of *Enterobacteriaceae* is their ability to ferment glucose, producing acid and gas, and their oxidase-negative status, which aids in laboratory identification. Importantly, several species are lactose fermenters, allowing rapid detection in clinical microbiology (Gautam et al., 2026).

Emerging data suggest that an increased relative abundance of *Enterobacteriaceae* is associated with gut inflammation, endotoxemia, and metabolic dysregulation. These bacteria possess lipopolysaccharide (LPS) in their outer membrane, a potent endotoxin that can trigger Toll-like receptor 4 (TLR4)-mediated inflammatory responses. Chronic low-grade endotoxemia induced by LPS has been proposed as a mechanistic link between the altered prevalence of culturable *Enterobacteriaceae* and obesity-related insulin resistance. Inflammatory signaling can impair hypothalamic leptin sensitivity, thereby contributing to leptin resistance (Singh et al., 2025). Furthermore, species such as adherent-invasive *Kpn* have been implicated in metabolic disturbances, suggesting a direct microbial contribution to adiposity and endocrine imbalance (Kumar et al., 2022).

The novelty of connecting *Enterobacteriaceae* with leptin dysregulation lies in integrating microbiological and hormonal paradigms of obesity. While hyperleptinemia is well documented, fewer studies have examined whether specific enteric pathogens or shifts within *Enterobacteriaceae* populations correlate with altered leptin levels in obese individuals. It is plausible that increased colonization or overgrowth of pro-inflammatory *Enterobacteriaceae* may enhance systemic inflammation (Kumar et al., 2022) disrupt gut barrier integrity and modulate adipocyte-derived leptin secretion or signaling. Such interactions could create a bidirectional axis wherein obesity-related hormonal changes favor dysbiosis, and dysbiosis further exacerbates leptin resistance (Gautam et al., 2025).

Therefore, exploring the prevalence and characterization of *Enterobacteriaceae* (with a major focus on facultative anaerobes) in obese individuals, alongside detailed hormonal profiling of leptin, ghrelin, and insulin, may provide novel insights into the gut-endocrine axis in obesity. Understanding whether specific enteric bacterial patterns are associated with hyperleptinemia or leptin resistance could open new avenues for microbiota-targeted therapeutic strategies, including dietary modulation. By integrating microbial ecology with endocrine dysfunction, this study aims to contribute to a more comprehensive understanding of obesity pathophysiology, particularly regarding metabolic and inflammatory interactions mediated by *Enterobacteriaceae*.

## 2 Materials and methods

### 2.1 Study population, sampling procedures

A case-control observational study was conducted at the Department of Physiology, Institute of Medical Sciences, Banaras Hindu University, Varanasi, between January 2022 and May 2023. Participants were recruited from the outpatient department (OPD) by staff and students after obtaining written informed consent. The study protocol was reviewed and approved by the Institutional Ethics Committee of IMS-BHU (Ref. No. Dean/2022/EC/3333), ensuring adherence to established ethical standards.

Initially, 86 male participants aged 18–41 years were enrolled. After applying exclusion & inclusion criteria, 2 participants refused to enroll in this study following weight and height measurements, and 63 eligible subjects remained. Four additional individuals were excluded for refusing to provide blood samples, and 10 for refusing to provide stool samples. The final analysis, therefore, included 49 participants: 27 males with obesity (BMI >23 kg/m², according to WHO Asian-BMI criteria) and 22 age-matched normal-weight controls (BMI 18–22.9 kg/m²) (**Supplementary Figure 1**). Although the sample size was modest, it reflected the stringent eligibility criteria and participant compliance required for the simultaneous collection of clinical, hormonal, dietary, and microbiological data. Therefore, this investigation should be regarded as an exploratory study. While statistically significant associations were identified, the relatively small sample size may have limited statistical power, increased the possibility of type II errors, and restricted the generalizability of the findings. Larger, multicenter studies are warranted to confirm these observations (Hajishizari et al., 2022).

### 2.2 Inclusion and exclusion criteria

People between 18 and 41 years old with a BMI exceeding 23 kg/m² were included in the study based on WHO Asian-BMI criteria for obesity in the Asian population (Adamska-Patruno et al., 2018; Ahirwar and Mondal, 2019; Lim et al., 2017). Exclusion criteria included people who had experienced significant weight loss in the past six months, were enrolled in any weight-loss program, had undergone weight reduction surgery, had chronic systemic illnesses, suffered from gastrointestinal disorders, or were taking hormonal supplements/antibiotics.

### 2.3 Data collection

All participants in the study completed a questionnaire to capture a detailed clinical history, followed by a physical examination and an assessment of their dietary habits and routine (Hwalla and Jaafar, 2020). Dietary habits were assessed using a structured semi-quantitative questionnaire administered through direct interviews. Participants were asked about the habitual frequency of consumption of major food categories including fast foods, fried foods, refined carbohydrates, soft drinks, fruits, vegetables, home-cooked meals, meat products, and vitamin/mineral supplements over the preceding three months. Dietary variables were categorized according to frequency of intake and coded for correlation analysis with anthropometric and hormonal parameters. The questionnaire was designed to evaluate dietary patterns and meal behaviors rather than detailed caloric or macronutrient intake; therefore, precise energy consumption was not calculated. Participants’ heights were recorded barefoot, accurate to 0.1 cm, while their body weights were taken using a calibrated digital scale with a precision of 0.1 kg (Diwan et al., 2018).

### 2.4 Blood collection and serum assay

Morning at 09:00 am, blood samples (3 ml) were collected from participants via venipuncture using plain vacutainers. The serum was separated from the blood following centrifugation at 3000 rpm for 5 minutes at 4 °C. The obtained serum was stored at -20 °C and subsequently analyzed for hormone concentrations using commercially available assay kits (Diwan et al., 2018).

Commercially available ELISA kits (ImmunoTag, USA) were used to measure insulin, ghrelin, and leptin serum levels. The serum concentration of leptin was assessed by the Human Leptin ELISA Kit (Cat.No ITEH01559) of Geno Technology Inc. (USA). The serum concentration of Ghrelin was evaluated by the Human Ghrelin ELISA Kit (Cat.No ITEH03091) of Geno Technology Inc. (USA). The serum concentration of Insulin was assessed by the Human Insulin ELISA Kit (Cat.No ITEH00010) of Geno Technology Inc. (USA). This sandwich kit is for accurate quantitative detection of Human leptin/ghrelin/insulin in serum, plasma, cell culture supernatants, ascites, tissue homogenates, or other biological fluids. The given ImmunoTag ELISA Kit Protocol was strictly followed.

An *in vitro* sandwich Enzyme-Linked Immunosorbent Assay (ELISA) (Geno Technology Inc., USA) was employed to quantitatively measure human leptin, ghrelin, and insulin in serum. A monoclonal antibody against leptin, ghrelin, and insulin was coated to the microtiter wells. Test and control samples were incubated in the coated wells with a specific anti-leptin/ghrelin/insulin antibody. After incubation, the unbound material was washed off, and horseradish peroxidase (HRP) conjugate was added to detect the bound leptin/ghrelin/insulin. The amount of leptin, ghrelin, and insulin in the sample correlated with the color intensity that developed (Al Maskari and Alnaqdy, 2006).

### 2.5 Stool sample collection and processing for bacterial isolation

Stool samples were collected from participants in Sterile Clinicol (HIMEDIA PW015) clean, dry, and leakproof sterile bottles. The specimens were immediately transported to the Microbiology Enteric Laboratory in the Department of Microbiology, Institute of Medical Sciences, and refrigerated at 4 °C prior to bacterial culture and identification.

A sterile wire loop was dipped into the stool sample to obtain an inoculum, which was streaked onto the surface of the plate containing MacConkey agar and Deoxycholate Citrate agar using the standard method (Kumar et al., 2022; Singh et al., 2025). The procedure was applied to each sample, and the plates were incubated at 37 °C for 24 hours. Each isolate’s presumed colonies on agar plates were further subcultured to obtain a pure culture. Pure isolates were kept for further bacterial identification.

### 2.6 Genomic fingerprinting of clinical isolates

Clinical Isolates were collected from stool samples of obese individuals and normal weight controls, and were identified to the species level utilizing standard microbiological, biochemical tests, and molecular techniques (Vishwakarma et al., 2025b). The E. coli strain ATCC 25922 was used as a reference control to accurately identify the isolates. *E. coli* & *Kpn* strains underwent fingerprinting using ERIC-PCR with primers described by Versalovic et al. (1991) (Versalovic et al., 1991). ERIC-PCR was used exclusively for molecular fingerprinting/strain typing (F: 5’ ATGTAAGCTCCTGGGGATTCAC-3’; R: 5’ AAGTAAGTGACTGGGGTGAGCG-3’), following established thermal cycling protocols. This involved an initial denaturation at 95 °C for 5 minutes, followed by 35 cycles of 10 seconds at 96 °C for denaturation, 30 seconds at 38.9 °C for annealing, and 1 minute at 72 °C for extension. The final extension step was completed at 72 °C for 10 minutes. The PCR products were analyzed using 1% agarose gel electrophoresis with a GeNei™ system (Sl. No-07/19/F/328, Peenya, Bangalore, India).

### 2.7 Scanning electron microscope

The morphology of *E. coli* & *Kpn* on GLA (glass cover slip) was assessed by SEM. Samples were fixed in 2.5% glutaraldehyde in PB buffer pH 7.2 for 10 min, dehydrated through an ethanol series (30, 50, 70, 90, and 100% v/v), and air-dried for 2 h. Surfaces underwent the same dehydration. All samples were dried for 1 day and sputter-coated with gold. Both surfaces and biofilm samples were viewed using a FESEM system (Zeiss GeminiSEM 500) at 7.00 kV. Cell morphology was measured using microscope software (xTMicroscope Control, FEI Company) (Vishwakarma et al., 2025b).

### 2.8 Statistical analysis

Data were analyzed using Excel and SPSS 25.0 and presented as mean ± standard deviation. The Mann-Whitney U test was used to assess statistical significance at a significance level of p < 0.05. Furthermore, Bonferroni multiple comparisons and correlations were conducted to examine the relationships among age, weight, height, BMI, dietary patterns, and leptin, ghrelin, and insulin levels in both groups.

## 3 Results

This study included 27 obese individuals and 22 age & height-matched controls (**Supplementary Figure 1**). The mean body weight of the obese was 81.04 ± 10.9 kg, which was considerably greater than that of the controls, 58.86 ± 5.962 kg. Similarly, the BMI in the study group was significantly higher at 28.644 ± 2.52 kg/m² than in the control group at 20.50 ± 1.997 kg/m² (**Table 1**).

**Table 1:** Comparison between characteristics of obese and controls.

Character	Controls (n=22) (Mean ± SD)	Obese (n=27) (Mean ± SD)	*p-value
Age (year)	26.32 ± 6.129	27.81 ± 2.988	0.071
Height (cm)	169.93 ± 7.680	167.941 ± 6.736	0.247
Weight (Kg)	58.86 ± 5.962	81.04 ± 10.983	0.001
BMI (Kg/m^2^)	20.50 ± 1.997	28.644 ± 2.52	0.001
Leptin (ng/mL)	3.683 ± 0.399	5.433 ± 0.677	0.001
Ghrelin (ng/mL)	3.522 ± 0.350	3.455 ± 0.279	0.778
Insulin (mIU/L)	2.494 ± 0.738	3.211 ± 0.276	0.003

*p < 0.05 statistically significant.

**Figure 1:**
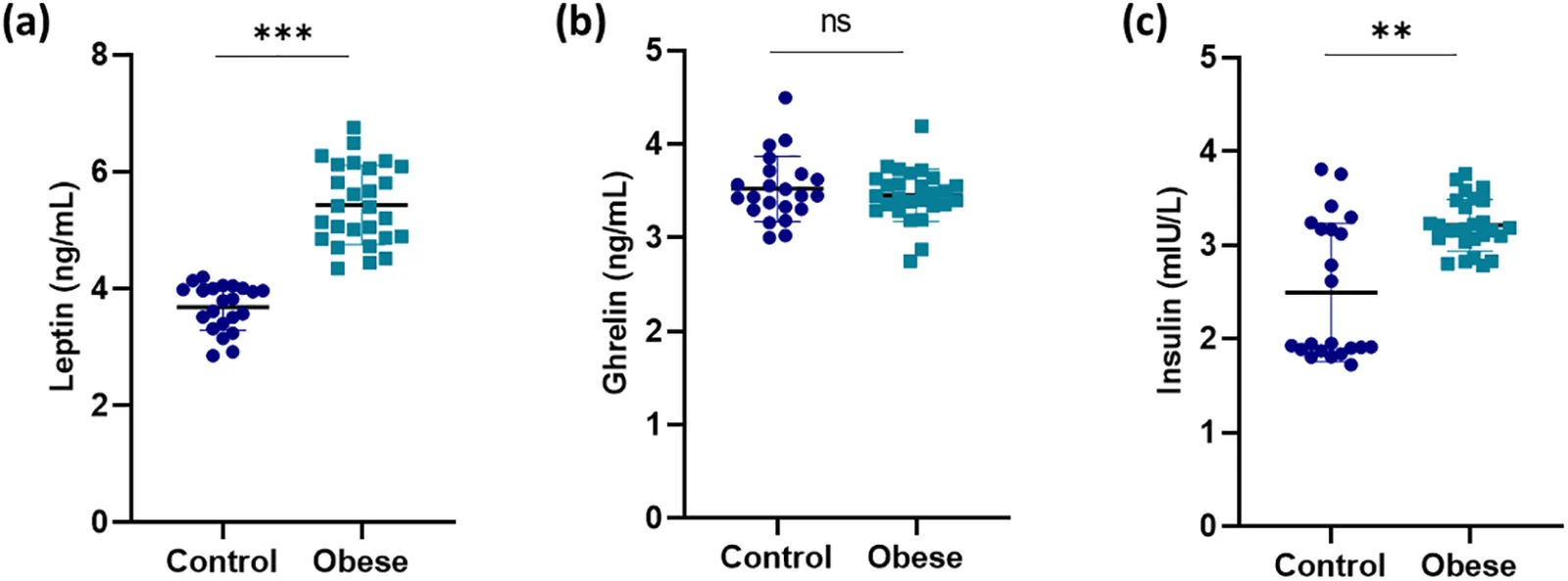
Serum concentrations of **(A)** leptin, **(B)** ghrelin, and **(C)** insulin in control and obese groups. ns- not significant, **p < 0.01; ***p < 0.001].

In obese individuals, the mean fasting serum leptin concentration was 5.433 ± 0.677 ng/ml, significantly higher than the mean of 3.683 ± 0.399 ng/ml observed in controls (p=0.001). Conversely, ghrelin levels were not significantly different between obese and controls (p=0.778). Insulin levels in obese were significantly higher at 3.211 ± 0.276 mUI/L, compared to 2.494 ± 0.738 mUI/L in the controls (p=0.003) (**Table 1** and **Figure 1**).

### 3.1 Dietary data shows notable differences between obese and control people

**Figure 2:**
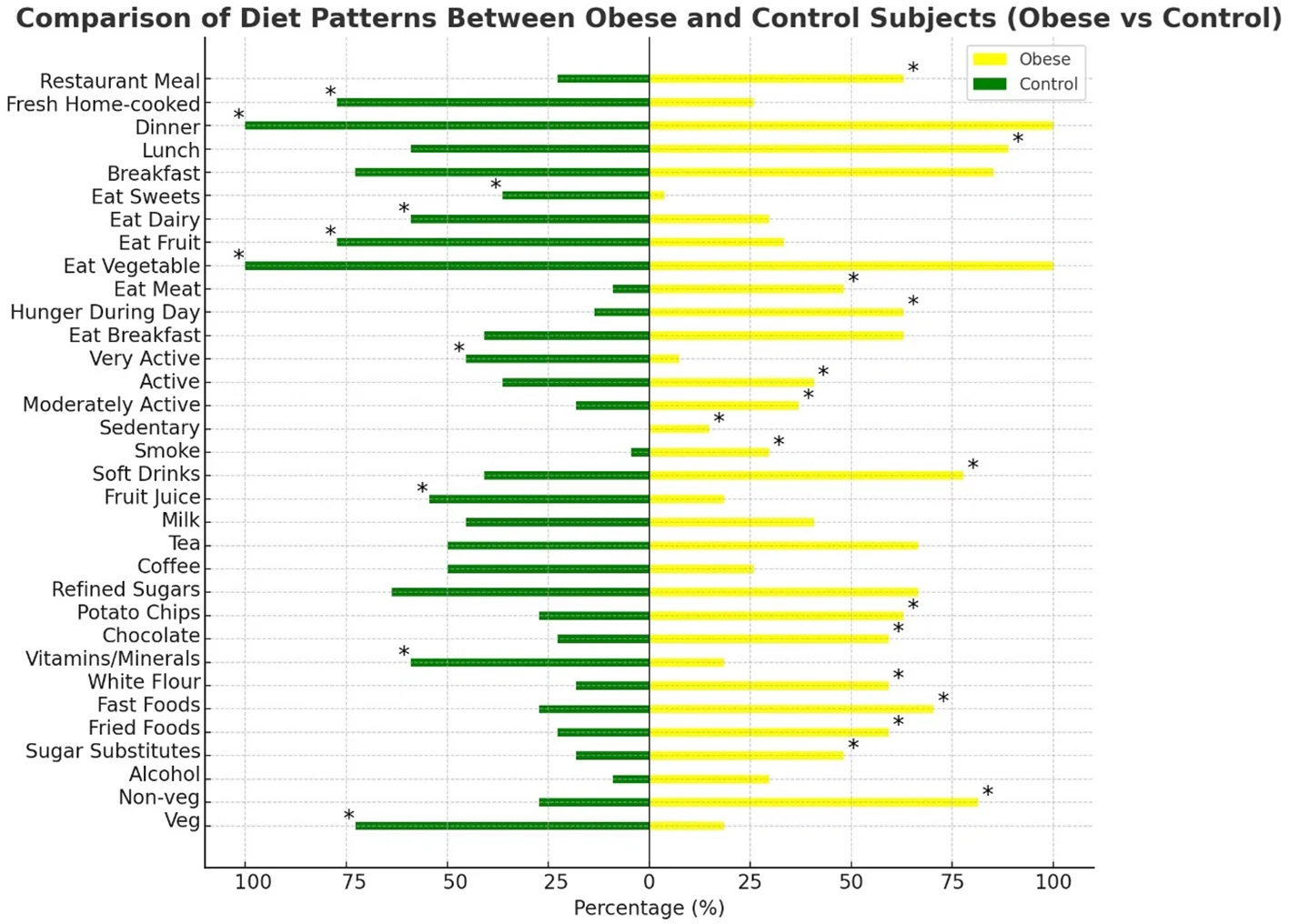
Comparison of dietary patterns and routines between obese and controls. *p-value ≤ 0.05 is significant.

Obese tend to consume more fast food (p= 0.002), fried foods (p= 0.009), white flour (p= 0.003), soft drinks (p= 0.008), and potato chips (p= 0.012), while intake of refined sugars was not significantly different from controls (p= 0.829). Obese gave a history of reduced intake of vegetables and freshly prepared home-cooked food. Additionally, they are more likely to eat a substantial breakfast, experience daytime hunger (p= 0.001), and have higher intake of meat (p= 0.003) compared to controls. However, they are less inclined to consume vitamins/minerals, fresh fruit juice, and fruits. Control group exhibit a higher intake of freshly prepared home-cooked food (p= 0.001), vegetables, fresh fruit juice (p= 0.008), fruits (p= 0.002), and vitamins/minerals (p= 0.003) and have history of lower consumption of fast food, fried foods, white flour, refined sugars, soft drinks along with a lower preponderance to experience day time hunger and meat consumption (**Supplementary Table 1**; **Figure 2**).

Serum biomarker levels were measured in all participants using blood samples collected after an overnight fast. A significant positive correlation (r = 0.423, p = 0.028) was found between serum leptin levels and BMI in obese individuals, but not in controls, suggesting that leptin levels tend to rise with increasing BMI (**Supplementary Figures 2A, B**). In obese individuals, no correlation existed between BMI and ghrelin levels. Conversely, the control group showed a significant negative correlation (r = -0.635, p = 0.001), indicating that controls have higher ghrelin levels (**Supplementary Figures 2C, D**). The control group (r = 0.224, p = 0.317) and the obese (r = 0.219, p = 0.273) both showed a positive correlation between insulin and BMI. However, the correlation was less pronounced in the controls (**Supplementary Figures 2E, F**). In contrast, the controls only showed a strong positive correlation between insulin and ghrelin (r = 0.624, p = 0.002) (**Supplementary Figures 2G, H**) as shown in **Table 2**.

**Table 2:** Correlation between serum leptin, ghrelin, and insulin concentration with BMI, weight, and leptin, ghrelin, and insulin.

Correlation	Obese (n=27)		Controls (n=22)	
	*r*	*p*	*r*	*p*
Leptin vs BMI	0.423^*^	0.028	-0.029	0.898
Ghrelin vs BMI	0.254	0.200	-0.635^**^	0.001
Insulin vs BMI	0.219	0.273	0.224	0.317
Leptin vs weight	0.159	0.429	-0.293	0.185
Ghrelin vs weight	0.064	0.752	0.644^**^	0.001
Insulin vs weight	0.298	0.131	0.429^*^	0.047
Leptin vs ghrelin	0.309	0.117	-0.194	0.387
Ghrelin vs insulin	0.206	0.301	0.624^**^	0.002
Insulin vs leptin	0.123	0.539	-0.215	0.337

*Correlation is significant at 0.05 (2-tailed).

**Correlation is significant at 0.01 level (2-tailed)

### 3.2 Dietary patterns correlate with hormones

**Figure 3:**
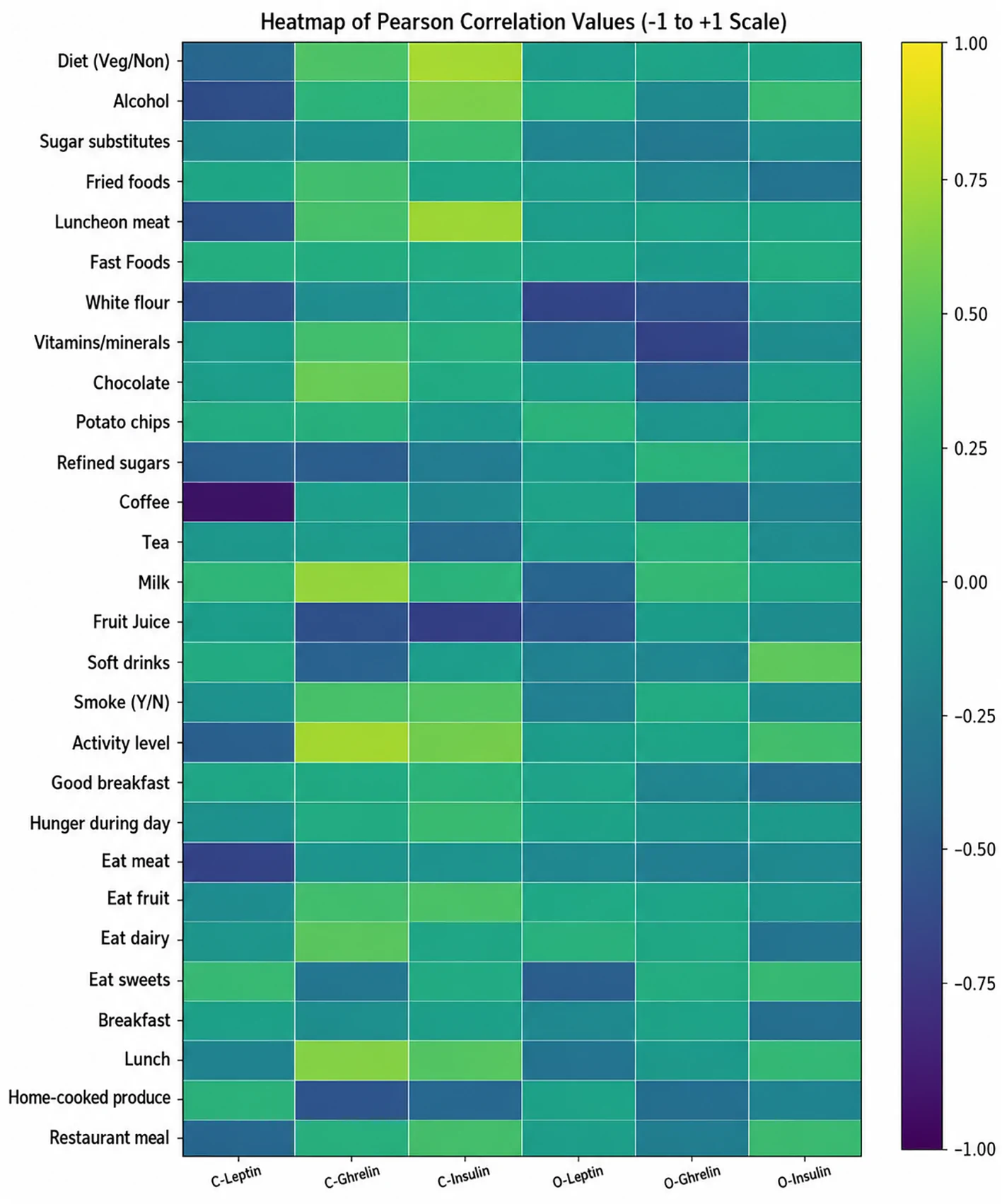
Heatmap showing correlation between dietary patterns and metabolic hormones in control and obese groups. The heatmap illustrates the strength and direction of correlations (r) between various dietary factors and serum leptin, ghrelin, and insulin levels in control (C) and obese (O) subjects. Warmer colors indicate positive correlations, whereas cooler colors signify negative correlations. Correlation coefficients range from −1 to +1, indicating inverse to direct associations, respectively. Statistically significant correlations (*p* < 0.05; p < 0.01) are detailed in the corresponding correlation table (**Supplementary Table 2**).

Controls: Leptin shows no significant connections to dietary factors. Ghrelin shows a strong negative correlation with freshly home-cooked produce (r = -0.476, p < 0.05). This indicates that those who eat more home-cooked meals tend to have reduced ghrelin levels. Insulin demonstrates a significant positive correlation with refined sugars (r = 0.336, p < 0.05), suggesting that higher consumption of refined sugars is linked to increased insulin levels (**Supplementary Table 2**; **Figure 3**).

Obesity: Leptin showed a significant negative correlation with freshly prepared home-cooked meals (**Supplementary Table 2**). Ghrelin exhibited a significant negative correlation with vitamin and mineral intake (r = −0.428, *p* < 0.05), whereas its association with freshly prepared home-cooked meals was not statistically significant (r = −0.231, *p* = 0.246). Insulin showed a significant positive correlation with refined sugar intake (**Supplementary Table 2**), while no significant correlations were observed with lunch or other dietary variables. Results are fully consistent with the correlation coefficients and p-values shown in **Supplementary Table 2**; **Figure 3**.

### 3.3 Bacterial isolation and processing

**Figure 4:**
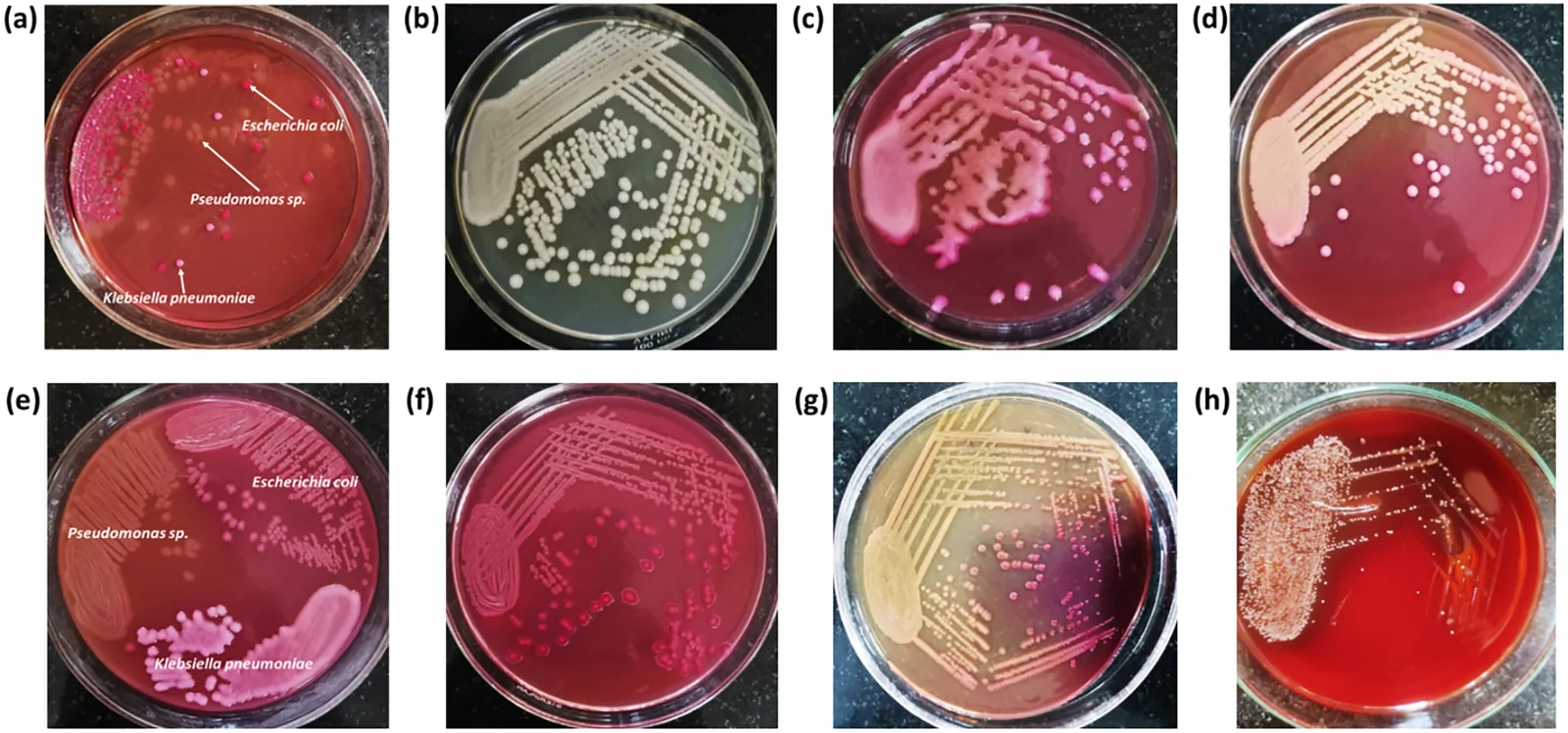
**(A)** Stool sample culture on MacConkey agar; **(B–D)** *Kpn* culture on different media; **(E)** bacterial isolates subculture; **(F–H)** *E. coli* culture on different media.

The cultural characteristics of the recovered isolates on MacConkey and Deoxycholate citrate agar are shown in **Figures 4A, E**. The results demonstrated that the recovered isolate was identified based on colony morphology and staining features. All the isolates were identified as Gram-negative rods, appearing as small, pink-colored rods that were either single or paired under the microscope, as shown in supplementary **Supplementary Figure 3A**. SEM images show *Kpn* in **Supplementary Figure 3B** and *E. coli* in **Supplementary Figure 3(C)**. The colorless colony with the jagged edge, clear colorless colony, mucoid pink colony, non-mucoid deeper pink colony, and pale-yellow colony are among the cultural characteristics. **Figures 4B–D** shows *Kpn*, while **Figures 4F–H** illustrates *E. coli*. **Table 3** shows the biochemical tests of different bacterial isolates.

**Table 3:** *Facultative anaerobic* bacteria isolated from the stool of obese and control individuals.

Organism name	Biochemical test											
	TSIA	SIM	Glu	Lac	Suc	Mann	Ureas	Citrate	Indole	H_2_S	Oxi	Cata
*Escherichia coli*	A/A	+ve	+ve	+ve	*v*	+ve	-ve	-ve	+ve	-ve	-ve	+ve
*Klebsiella pneumoniae*	A/A	-ve	+ve	+ve	+ve	+ve	+ve	+ve	-ve	-ve	-ve	+ve
*Citrobacter* spp.	A/A_black_	+ve	+ve	+ve	+ve	+ve	*v*	+ve	-ve	+ve	-ve	+ve
*Pseudomonas* spp.	K/K	+ve	-ve	-ve	-ve	+ve	-ve	+ve	-ve	-ve	+ve	+ve

TSIA, Triple Sugar Iron Agar, SIM, Sulphur Indole Motility, Glu, Glucose, Lac, Lactose, Suc, Sucrose, Mann, Mannitol, Oxi, Oxidase, Cata, Catalase, A/A, Acidic butt/Acidic slant, K/K,Alkaline butt/Alkaline slant, (+ve = positive), (-ve = negative), (*v*= variable).

**Table 4** presents the distribution of bacterial isolates obtained from stool samples of obese and control participants. A total of 84 isolates were identified, of which 54 (100%) were recovered from the obese group and 39 (100%) from the control group.

**Table 4:** Clinical specimen isolates from stool samples from obese and control.

Isolates	Obese (n=27)	Control (n=22)	*p value*
*Escherichia coli*	20 (48.78%)	21 (51.22%)	>0.05
*Klebsiella pneumoniae*	26 (92.88%)	2 (7.14%)	<0.001
*Pseudomonas sp*	4 (50%)	4 (50%)	>0.05
*Citrobacter sp*	3 (60%)	2 (40%)	>0.05
Total *(n=84)*	*53 (64.63%)*	*28 (35.37%)*	

**Figure 5:**
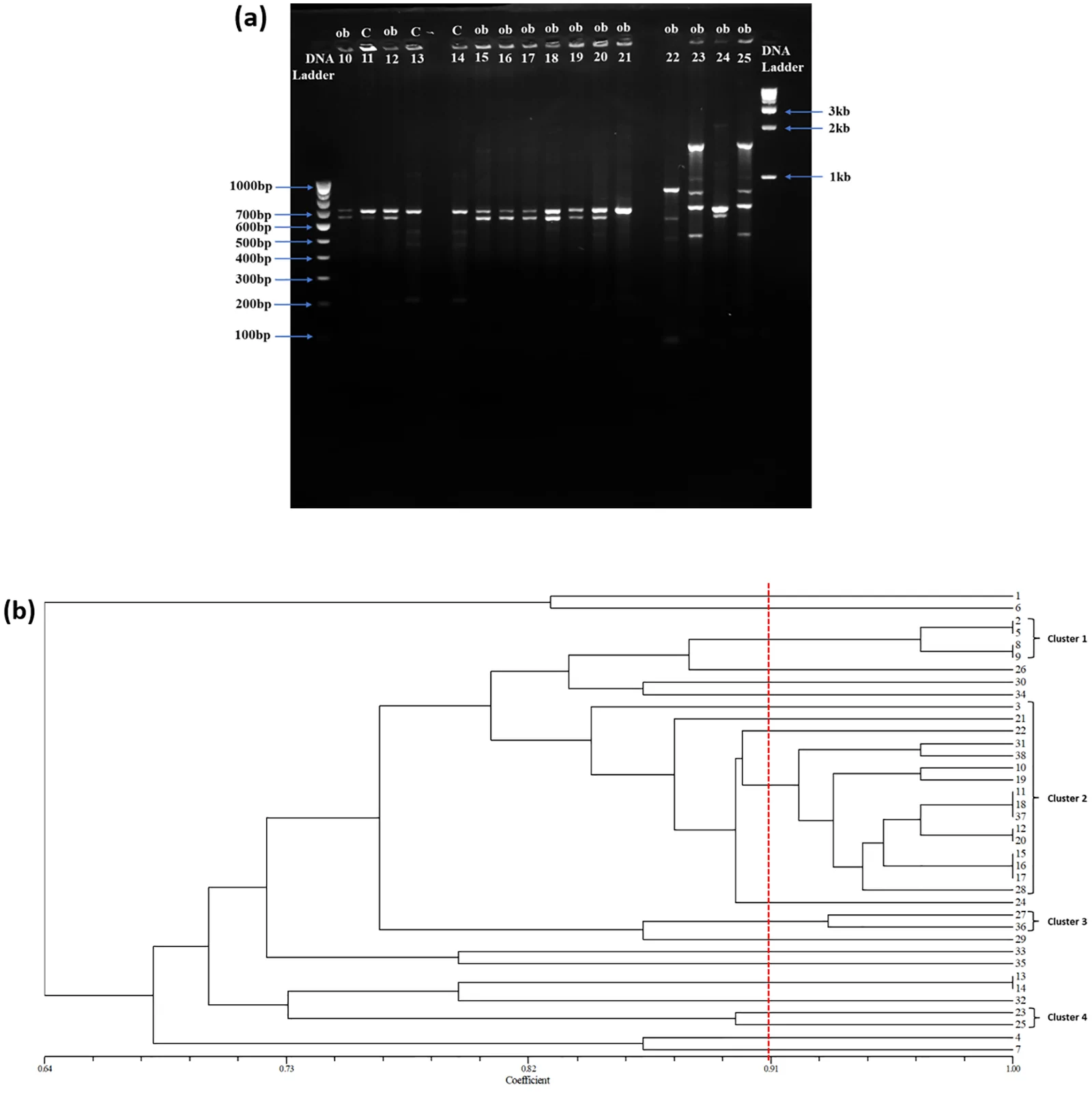
**(A)** Genomic fingerprinting: PCR amplification of *Kpn* genomic DNA using ERIC primer, **(B)** Dendrogram obtained by ERIC-PCR of strains.

**Figure 6:**
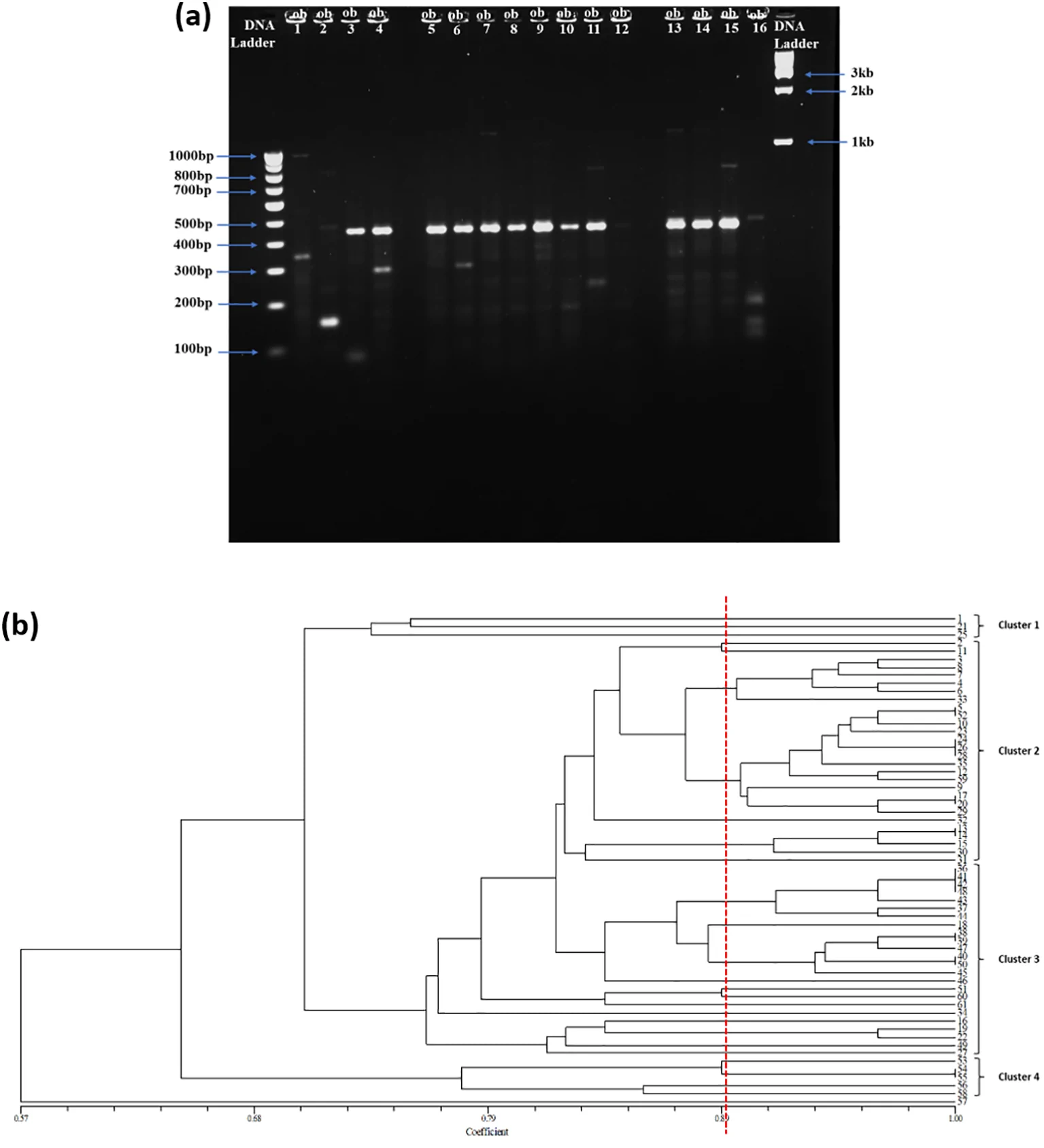
**(A)** Genomic fingerprinting: PCR amplification of *E. coli* genomic DNA using ERIC primer. **(B)** Dendrogram obtained by ERIC-PCR of strains.

Gel electrophoresis analysis of Enterobacterial Repetitive Intergenic Consensus-Polymerase Chain Reaction (ERIC-PCR) fingerprinting of *E. coli* and *Kpn* strains isolated from control and obese stool samples. Phylogenetic tree based on the ERIC-PCR data showing the relationships among all 41 *E. coli* and 28 *Kpn* strains studied. Clusters were determined using the unweighted pair group method with arithmetic mean (UPGMA) method and the Dice similarity coefficient. The banding pattern revealed 4 distinct clusters and 11 singletons in **Figures 5A, B**. Cluster 2 had the highest prevalence (48%) and contained the majority of *Kpn* strains from obese individuals. This suggests that a specific strain of *Kpn* might be more closely linked to, or potentially associated with obesity. The banding pattern of *E. coli* revealed four distinct clusters and one singleton in **Figures 6A, B**. Clusters 2 and 3 had the highest prevalence.

## 4 Discussion

The present study explored the complex interplay between metabolic hormones, dietary habits, and enteric bacterial colonization in obese and normal-weight adult males. By integrating endocrine profiling with microbiological assessment, our findings provide a multidimensional perspective on obesity that extends beyond traditional energy imbalance models. In particular, the predominance of *Enterobacteriaceae*, especially *Kpn* in obese individuals highlights a potentially important microbial contribution to hormonal dysregulation and metabolic inflammation (Ispas et al., 2023).

### 4.1 Hormonal dysregulation in obesity

Consistent with established literature, obese participants in our study exhibited significantly elevated fasting serum leptin and insulin levels compared to controls (Al-Sultan and Al-Elq, 2006; Korek et al., 2013). Hyperleptinemia reflects increased adipose tissue mass, as leptin is secreted proportionally to fat stores. However, despite high circulating levels, appetite suppression fails in obesity due to leptin resistance a condition attributed to impaired hypothalamic signaling, reduced leptin receptor (LEPR/OB-R) sensitivity, and defective transport across the blood–brain barrier (Caro et al., 1996; Korek et al., 2013). The significant positive correlation between leptin and BMI observed in obese participants further supports the notion that increasing adiposity drives greater leptin production without restoring energy balance (Martins et al., 2012).

Similarly, fasting insulin levels were significantly higher in obese subjects, suggesting compensatory hyperinsulinemia caused by insulin resistance (Caricilli and Saad, 2013; Hardy et al., 2012). Chronic low-grade inflammation, often associated with obesity, contributes to impaired insulin receptor signaling and glucose uptake. The interaction between leptin and insulin is well recognized; hyperinsulinemia can enhance leptin secretion, while leptin resistance may further exacerbate insulin resistance, creating a self-perpetuating metabolic cycle (Caricilli and Saad, 2013).

In contrast, fasting ghrelin levels did not differ significantly between the obese and control groups, consistent with previous finding (Votruba et al., 2009). However, the lack of a correlation between ghrelin and BMI in the obese group, unlike the strong positive correlation seen in controls, indicates a disruption in normal appetite signaling associated with obesity. A previous study reported that ghrelin concentrations in lean girls show diurnal variability, with increases before breakfast and dinner, consistent with our study, which collected blood samples at 9:00 am (Foster et al., 2007). The preserved ghrelin-BMI association in controls likely reflects intact physiological regulation, whereas the breakdown of this relationship in obese individuals indicates endocrine dysregulation at higher BMI thresholds.

### 4.2 *Enterobacteriaceae* and obesity: a microbial perspective

Beyond hormonal imbalance, our microbiological analysis revealed a notable predominance of *Enterobacteriaceae* in stool samples from obese individuals. This family, belonging to the phylum Proteobacteria (Vishwakarma et al., 2026), comprises Gram-negative, facultatively anaerobic bacilli commonly residing in the human gut. While many are commensals, several species act as opportunistic pathogens and have been implicated in metabolic disorders (Rizzatti et al., 2017).

Among these, *Kpn* was predominantly isolated in obese individuals. This observation aligns with previous findings suggesting an association between *Klebsiella* overgrowth and weight gain in an animal model (Kumar et al., 2022). *Kpn* is known for its capacity to produce endotoxins (lipopolysaccharides, LPS), which can translocate across a compromised intestinal barrier, triggering systemic inflammation. Chronic endotoxemia has been proposed as a mechanistic link between altered prevalence of culturable *Enterobacteriaceae* and obesity (Cani et al., 2007; Rizzatti et al., 2017). LPS activates Toll-like receptor 4 (TLR4)-mediated pathways, leading to increased production of pro-inflammatory cytokines such as TNF-α and IL-6, both of which are implicated in insulin resistance and leptin signaling impairment (Cani et al., 2008; Gautam et al., 2026).

The predominance of *Kpn* in obese subjects in our cohort may therefore contribute to leptin resistance through inflammatory mechanisms. Elevated circulating LPS levels can disrupt hypothalamic leptin signaling pathways, reducing receptor sensitivity despite hyperleptinemia (Cani et al., 2008; Rizzatti et al., 2017). This possible associations between Enterobacteriaceae prevalence and metabolic hormone alterations interaction offers a plausible explanation for the persistent hunger and metabolic imbalance observed in obesity. Furthermore, *Kpn* species have demonstrated enhanced energy-harvesting capabilities from dietary substrates, potentially increasing caloric extraction and fat deposition (Kumar et al., 2022). Other *Enterobacteriaceae* members, including *Escherichia coli, Salmonella, Shigella, Citrobacter*, and *Enterobacter cloacae*, were also identified. *Enterobacter cloacae* has been experimentally associated with the development of obesity in both animal and human studies, potentially via endotoxin production and activation of inflammation (Fei and Zhao, 2013).

### 4.3 Correlation of *Klebsiella* with BMI and hormonal markers

A particularly noteworthy finding in our study was a positive association between *Kpn* and higher BMI. Obese individuals with *Kpn* showed tendencies toward higher leptin and insulin levels compared to control participants without predominant *Kpn* growth (Kumar et al., 2022). Although causality cannot be established in this cross-sectional design, the correlation suggests a possible synergistic effect between microbial dysbiosis and endocrine disruption.

The coexistence of hyperleptinemia, hyperinsulinemia, and *Kpn* dominance may suggest that microbial inflammation heightens metabolic resistance pathways (Gong et al., 2020). Persistent exposure to endotoxins could maintain systemic inflammatory tone, exacerbating leptin receptor desensitization and impairing insulin signaling cascades (Cani et al., 2007; Kumar et al., 2022). Thus, the gut microbiota may not only reflect obesity-related changes but also actively contribute to metabolic deterioration.

### 4.4 Dietary patterns and microbial-hormonal interactions

Dietary assessment in our cohort revealed higher consumption of fast foods, fried foods, refined sugars, white flour products, and soft drinks among obese individuals. Such diets are known to favor the proliferation of Proteobacteria, including *Enterobacteriaceae* (Saha and Thapar, 2025). High-fat and high-sugar diets alter gut permeability and microbial diversity, potentially promoting *Kpn* overgrowth (Kumar et al., 2022). Significant correlations between refined sugar intake and insulin levels in both groups support the established link between dietary glycemic load and hyperinsulinemia. Additionally, the negative correlation between ghrelin levels and freshly home-cooked produce suggests that diets rich in fiber and micronutrients may beneficially modulate appetite-regulating hormones. The reduced intake of fruits and vegetables among obese participants may therefore not only influence endocrine balance but also promote microbial dysbiosis. The observed pattern indicates a triadic interaction: an unhealthy diet fosters *Enterobacteriaceae* overgrowth, which induces inflammatory responses and subsequently impairs leptin and insulin signaling (Rizzatti et al., 2017; Saha and Thapar, 2025). This integrated model strengthens the hypothesis that obesity is not merely an endocrine disorder but a microbiota-associated inflammatory state.

### 4.5 Limitations

This study has several limitations that should be considered when interpreting the findings. First, the relatively small sample size reduces statistical power and limits the generalizability of the findings, precluding robust causal inference. Second, its cross-sectional design prevents determination of temporal relationships between *Klebsiella pneumoniae* predominance and leptin–insulin dysregulation, Unfortunately, fasting glucose measurements were not available; therefore, HOMA-IR calculation could not be performed. Third, an important limitation of the present study is that the microbiological analysis was restricted to selective culture-based identification of facultative anaerobic Enterobacteriaceae. This methodology does not comprehensively represent total gut microbial diversity, particularly anaerobic and unculturable taxa that constitute a major proportion of the intestinal microbiota. Therefore, the findings should not be interpreted as a complete characterization of gut dysbiosis. In addition, culture-based methods may introduce selection bias toward easily cultivable organisms. Future studies incorporating 16S rRNA sequencing, metagenomics, and metabolomic profiling are required for comprehensive microbiome characterization and validation of the observed microbial-hormonal associations. Finally, the absence of sequencing-based microbiome profiling and inflammatory biomarker measurements constrains mechanistic interpretation. Mechanistic pathways involving LPS-TLR4-TNF-α/IL-6 are now explicitly described as hypothetical and literature-supported rather than demonstrated by our data.

The study also has limited external generalizability because it included only adult Indian males recruited from a single center. Sex-specific hormonal variations, regional dietary habits, and ethnic variability may influence obesity-associated microbial and endocrine profiles. Furthermore, dietary assessment was based on self-reported frequency questionnaires without detailed caloric or macronutrient quantification, which may introduce recall bias and limit nutritional precision.

## 5 Conclusion

In summary, our findings suggest that obesity in this cohort of males is characterized not only by hyperleptinemia and hyperinsulinemia but also by a predominance of *Enterobacteriaceae*, particularly *Klebsiella pneumoniae*. The correlation between *Klebsiella* presence, higher BMI, and altered hormonal profiles supports a potential possible associations between *Enterobacteriaceae* prevalence and hormonal alterations contributing to metabolic dysregulation. These results underscore the importance of integrating microbial analysis with hormonal and dietary assessment to better understand obesity pathophysiology and to develop targeted therapeutic strategies addressing both endocrine imbalance and altered prevalence of culturable *Enterobacteriaceae*. These findings should be interpreted within the context of adult Indian males from a single-center cohort and require validation in larger, geographically and demographically diverse populations.

## Data Availability

The original contributions presented in the study are included in the article/**Supplementary Material**. Further inquiries can be directed to the corresponding author.
