# Supplementary material for: Integrated microbial and endocrine signatures of obesity: Enterobacteriaceae alterations, hormonal profiles, and dietary correlates in adult males
Source: Front Microbiomes. 2026 Sep 9;5:1867961. doi: 10.3389/frmbi.2026.1867961 (PMC13597868; doi:10.3389/frmbi.2026.1867961)
Supplement: Supplementary file 1 [file Supplementaryfile1.docx]

**Integrated Microbial and Endocrine Signatures of Obesity: Enterobacteriaceae Alterations, Hormonal Profiles, and Dietary Correlates in Adult Males**

Ranjeet Kumar Vishwakarma^1^, *Bhupendra Singh Yadav^1^, Priyanka Gautam^2^, Minakshi Sahu^3^, Gopal Nath^3^

**Fig. S1:** Summary diagram of recruitment process by Exclusion and Inclusion criteria

**Table S1:** One-Way ANOVA Comparing Dietary and Lifestyle Variables Between Groups (N = 49)

| **Variable** | ***p-value*** |
| --- | --- |
| Diet (Veg/Non) | <0.001 |
| Alcohol | 0.079 |
| Sugar substitutes (Nutra-Sweet) | 0.028 |
| Fried foods | 0.009 |
| Luncheon meat | <0.001 |
| Fast Foods | 0.002 |
| White flour | 0.003 |
| Margarine | - |
| Vitamins/minerals | 0.003 |
| Chocolate | 0.009 |
| Potato chips | 0.012 |
| Refined sugars | 0.829 |
| Coffee | 0.085 |
| Tea | 0.247 |
| Milk | 0.746 |
| Fruit Juice | 0.008 |
| Soft drinks | 0.008 |
| Vegetable Juice | - |
| Smoke (Y/N) | 0.024 |
| Activity level (Grade) | <0.001 |
| Eat a good breakfast | 0.129 |
| Feeling of hunger during the day | <0.001 |
| Eat meat | 0.003 |
| Eat vegetable | - |
| Eat fruit | 0.002 |
| Eat dairy | 0.039 |
| Eat sweets | 0.003 |
| Breakfast | 0.292 |
| Lunch | 0.015 |
| Dinner | - |
| Freshly home-cooked produce | <0.001 |
| Restaurant meal | 0.004 |
| Avoid food due to health issue | - |
| Food allergy | - |

**Fig S2:** The relationship of serum biomarkers to BMI and interactions between leptin, ghrelin, & insulin in both obese and control subjects include: (a, b) the correlation between leptin levels and BMI, (c, d) the correlation between ghrelin levels and BMI, (e, f) the correlation between insulin levels and BMI, and (g, h) the relationship between insulin levels and ghrelin.

**Table S2. Correlation Between Dietary Patterns and Leptin, Ghrelin, & Insulin Levels in people with obesity vs Controls**

|  | **Controls** | | | | | | **People with obesity** | | | | | | |
| --- | --- | --- | --- | --- | --- | --- | --- | --- | --- | --- | --- | --- | --- |
| **Dietary Pattern** | **Leptin (ng/mL)** |  | **Ghrelin (ng/mL)** |  | **Insulin (mIU/L)** |  | **Leptin (ng/mL)** |  | **Ghrelin (ng/mL)** |  | **Insulin (mIU/L)** |  |  |
|  | **Pearson Correlation** | **Sig. (2-tailed)** | **Pearson Correlation** | **Sig. (2-tailed)** | **Pearson Correlation** | **Sig. (2-tailed)** | **Pearson Correlation** | **Sig. (2-tailed)** | **Pearson Correlation** | **Sig. (2-tailed)** | **Pearson Correlation** | **Sig. (2-tailed)** |  |
| **Diet (Veg/Non)** | -0.305 | 0.168 | 0.305 | 0.167 | .520^*^ | 0.013 | 0.005 | 0.979 | 0.040 | 0.843 | 0.085 | 0.673 |  |
| **Alcohol** | -0.379 | 0.082 | 0.215 | 0.337 | .436^*^ | 0.043 | 0.081 | 0.689 | -0.130 | 0.519 | 0.218 | 0.275 |  |
| **Sugar substitutes (Nutra-Sweet)** | -0.114 | 0.615 | -0.055 | 0.807 | 0.256 | 0.250 | -0.118 | 0.557 | -0.161 | 0.421 | -0.087 | 0.664 |  |
| **Fried foods** | 0.077 | 0.734 | 0.296 | 0.181 | 0.091 | 0.686 | -0.012 | 0.952 | -0.137 | 0.497 | -0.202 | 0.313 |  |
| **Luncheon meat** | -0.305 | 0.168 | 0.305 | 0.167 | .520^*^ | 0.013 | 0.005 | 0.979 | 0.040 | 0.843 | 0.085 | 0.673 |  |
| **Fast Foods** | 0.200 | 0.371 | 0.197 | 0.379 | 0.130 | 0.563 | 0.046 | 0.821 | 0.019 | 0.924 | 0.113 | 0.573 |  |
| **White flour** | -0.417 | 0.053 | -0.022 | 0.924 | 0.065 | 0.775 | -.440^*^ | 0.022 | -0.380 | 0.050 | -0.006 | 0.976 |  |
| **Vitamins/minerals** | 0.014 | 0.949 | 0.308 | 0.163 | 0.143 | 0.527 | -0.325 | 0.098 | -.428^*^ | 0.026 | -0.068 | 0.737 |  |
| **Chocolate** | 0.030 | 0.896 | .437^*^ | 0.042 | 0.129 | 0.567 | 0.021 | 0.916 | -0.258 | 0.194 | 0.015 | 0.942 |  |
| **Potato chips** | 0.112 | 0.621 | 0.220 | 0.324 | -0.026 | 0.909 | 0.170 | 0.397 | -0.063 | 0.756 | 0.061 | 0.763 |  |
| **Refined sugars** | -0.336 | 0.126 | -0.374 | 0.087 | -0.220 | 0.326 | 0.024 | 0.905 | 0.171 | 0.393 | -0.057 | 0.777 |  |
| **Coffee** | -.550^**^ | 0.008 | 0.008 | 0.972 | -0.184 | 0.412 | 0.032 | 0.873 | -0.275 | 0.165 | -0.148 | 0.462 |  |
| **Tea** | 0.005 | 0.982 | -0.014 | 0.952 | -0.301 | 0.173 | 0.018 | 0.930 | 0.160 | 0.425 | -0.113 | 0.576 |  |
| **Milk** | 0.207 | 0.356 | .495^*^ | 0.019 | 0.112 | 0.619 | -0.281 | 0.155 | 0.236 | 0.235 | 0.014 | 0.943 |  |
| **Fruit Juice** | 0.020 | 0.931 | -0.393 | 0.070 | -.494^*^ | 0.019 | -.388^*^ | 0.046 | 0.001 | 0.996 | -0.081 | 0.689 |  |
| **Soft drinks** | 0.089 | 0.695 | -0.374 | 0.087 | 0.003 | 0.988 | -0.153 | 0.445 | -0.103 | 0.611 | 0.355 | 0.069 |  |
| **Smoke (Y/N)** | -0.095 | 0.674 | 0.298 | 0.178 | 0.397 | 0.067 | -0.165 | 0.411 | 0.147 | 0.464 | -0.085 | 0.674 |  |
| **Activity level (Grade)** | -0.333 | 0.130 | .571^**^ | 0.006 | .449^*^ | 0.036 | -0.013 | 0.948 | 0.058 | 0.773 | 0.300 | 0.128 |  |
| **eat a good breakfast** | 0.085 | 0.708 | 0.123 | 0.586 | 0.231 | 0.301 | 0.026 | 0.896 | -0.120 | 0.550 | -0.249 | 0.211 |  |
| **feeling of hunger during the day** | -0.132 | 0.559 | 0.127 | 0.573 | 0.256 | 0.251 | 0.034 | 0.867 | -0.048 | 0.811 | -0.028 | 0.888 |  |
| **eat meat** | -.485^*^ | 0.022 | 0.013 | 0.952 | 0.004 | 0.986 | -0.118 | 0.557 | -0.161 | 0.421 | -0.087 | 0.664 |  |
| **eat fruit** | -0.071 | 0.752 | 0.260 | 0.243 | 0.267 | 0.229 | 0.102 | 0.612 | 0.084 | 0.676 | -0.049 | 0.808 |  |
| **eat dairy** | -0.050 | 0.824 | 0.373 | 0.087 | 0.072 | 0.749 | 0.200 | 0.318 | 0.120 | 0.550 | -0.156 | 0.438 |  |
| **eat sweets** | 0.183 | 0.416 | -0.169 | 0.451 | 0.102 | 0.653 | -0.208 | 0.298 | 0.131 | 0.515 | 0.191 | 0.341 |  |
| **Breakfast** | -0.014 | 0.952 | -0.137 | 0.543 | 0.061 | 0.789 | -0.128 | 0.524 | 0.083 | 0.682 | -0.196 | 0.326 |  |
| **Lunch** | -0.138 | 0.541 | .450^*^ | 0.035 | 0.318 | 0.149 | -0.191 | 0.341 | -0.010 | 0.960 | 0.222 | 0.265 |  |
| **Freshly home-cooked meal** | 0.185 | 0.410 | -.475^*^ | 0.026 | -0.272 | 0.221 | 0.089 | 0.660 | -0.231 | 0.246 | -0.132 | 0.512 |  |
| **Restaurant meal** | -0.297 | 0.179 | 0.262 | 0.238 | 0.353 | 0.107 | 0.113 | 0.574 | -0.147 | 0.464 | 0.362 | 0.063 |  |

*. Correlation is significant at 0.05 (2-tailed)

**. Correlation is significant at 0.01 level (2-tailed)

**Fig S3:** (a) Gram staining, SEM microscopy of (b) *Klebsiella pneumoniae* and (c) *Escherichia coli*
